# Prevalence and Factors Associated with Repeat Mental Health Service Utilization During Rwanda’s Genocide Commemoration Week

**DOI:** 10.3390/ijerph22071019

**Published:** 2025-06-27

**Authors:** Anne Marie Bamukunde, Darius Gishoma, Bakang Percy Tlhaloganyang, Amparo Elena Gordillo-Tobar, Nancy Claire Misago, Claude Mambo Muvunyi

**Affiliations:** 1 Rwanda Biomedical Center, Kigali P.O. Box 7162, Rwanda; bamukunde78@gmail.com (A.M.B.); darius.gishoma@rbc.gov.rw (D.G.); nancy.misago@rbc.gov.rw (N.C.M.); claude.muvunyi@rbc.gov.rw (C.M.M.); 2 World Bank Group, Washington, DC 20433, USA; agordillotobar@worldbank.org

**Keywords:** repeat mental health service utilization, genocide commemoration week, Rwanda

## Abstract

The genocide commemoration week in Rwanda often triggers heightened mental health (MH) needs, necessitating targeted support. Understanding factors influencing repeat MH service utilization is essential for effective interventions. This cross-sectional study analyzed data from individuals seeking MH services during the 2024 Rwandan genocide commemoration week, distinguishing between first-time and repeat users. Descriptive and logistic regression analyses examined factors associated with repeat utilization. Of the 825 individuals who accessed MH services during Rwanda’s 2024 genocide memorial week, 76% were repeat users. Bivariate analysis showed that age and insurance coverage were significantly associated with repeat service utilization, while gender and province were not. Logistic regression revealed that individuals aged 31–50 (AOR = 2.29, 95% CI: 1.13–4.64, *p* = 0.022) and those without insurance coverage (AOR = 3.31, 95% CI: 1.78–6.18, *p* < 0.001) were more likely to be repeat users compared to the reference groups (18–30 years old and those with insurance, respectively). Gender and province remained nonsignificant in the adjusted model. Improving MH access, particularly for middle-aged individuals and the uninsured, is crucial. Addressing barriers to care could enhance service delivery during the commemoration period.

## 1. Introduction

The yearly commemoration of the Rwandan Genocide holds profound significance, both historically and psychologically, for the nation and its people. Following the atrocities of 1994, Rwanda has grappled with enduring MH challenges among survivors and their descendants [1,2]. The trauma inflicted during this dark period continues to permeate Rwandan society, influencing attitudes, behaviors, and access to MH services [1]. Given the lingering impact of the genocide, understanding how individuals utilize MH services becomes imperative, given that people are still wrestling with unresolved trauma or heightened emotional distress [3]. Investigating the factors that shape MH service utilization is not only essential for addressing ongoing healthcare needs but also for fostering healing and resilience within the Rwandan community [4].

Recent research highlights the profound and persistent MH challenges faced by Rwandans since the 1994 genocide, with studies revealing high rates of post-traumatic stress disorder (PTSD) and depression, the intergenerational transmission of trauma, and significant psychological burdens on both survivors and their descendants [5,6,7,8]. These studies underscore the need for targeted MH interventions, especially as trauma impacts vulnerable groups differently, with findings pointing to gender-specific symptoms, increased risk of suicide, and unique psychological effects on rape survivors [9,10,11,12]. Additional insights revealed the cultural and societal complexities of MH within Rwandan families, illustrating how trauma is transmitted across generations, often limiting reconciliation efforts [13,14,15]. Research on Rwandan refugees in the U.S. emphasizes the need for culturally sensitive MH services to address stigma and access barriers [16].

Despite these significant contributions, there remains a gap in understanding the specific factors driving repeat MH service utilization. Existing studies have largely focused on the general prevalence and impact of PTSD, depression, and other MH disorders, but few have examined the factors associated with repeated service utilization during periods of intense remembrance and potential traumatization. This study aims to investigate the frequency and factors driving repeat MH service utilization during the genocide commemoration week in Rwanda.

## 2. Materials and Methods

The dataset utilized in this study was collected by MH professionals during the official week of the 2024 Rwandan Genocide commemoration. Unlike previous years, when only aggregate data were recorded, the 2024 dataset represents the first systematic individual-level documentation of mental health (MH) service utilization during this period. This dataset was created specifically for research purposes and is not derived from an electronic health record system.

Individuals seeking MH support accessed services through various pathways, including self-referral, referral by family members or healthcare professionals, and home visits conducted by MH workers following reports from relatives or community members. The dataset primarily focuses on essential demographic and contextual variables relevant to client assistance and support during the commemoration period. These variables include information such as gender, age, geographic location of service, and insurance coverage status.

A key variable of interest was ‘MH service utilization’, which distinguishes between individuals seeking MH services for the first time during the genocide commemoration week and those who had previously accessed services during past genocide commemoration weeks. Repeat users in this study are defined strictly as individuals who sought services during previous commemoration periods, and this classification does not include individuals who use mental health services at other times of the year. The study does not track MH service utilization outside this specific period or in other consultation settings.

This study employed Stata 17 to conduct a comprehensive analysis of MH service utilization during the genocide commemoration week. MH service utilization was categorized as a binary variable with two levels: “first-time user” and “repeat user”. The analysis began with descriptive statistics, summarizing the frequencies and percentages of individuals utilizing MH services during this period. This initial analysis provided a foundational understanding of the distribution of service use across various demographic and contextual variables. Chi-square tests and logistic regression analyses were employed to assess the statistical significance of factors influencing MH service utilization. Crude odds ratios (COR) and adjusted odds ratios (AOR), along with their respective *p*-values and 95% confidence intervals (95%-CI), were estimated. Gender, age, province, and insurance coverage were included as independent variables in the logistic regression model.

## 3. Results

A total of 825 individuals accessed MH services during Rwanda’s 2024 genocide commemoration week. Of these, 76% (624 individuals) were identified as repeat users, while 24% (201 individuals) were first-time users, indicating a high prevalence of recurring MH needs during this period of remembrance.

Table 1 shows that a total of 825 participants were included, of whom 723 (87.6%) were female and 102 (12.4%) were male. The majority of participants were 31–50 years (57.3%), followed by those over 50 years (27.6%), while younger participants under 18 years and 18–30 years constituted smaller proportions (6.2% and 9.0%, respectively). Females were more represented across all age categories compared to males, except among those over 50 years, for who males accounted for 22.1% compared to 28.4% for females.

Geographically, most participants resided in Kigali City (33.4%), with slightly fewer participants from the Southern (24.7%), Northern/Western (20.6%), and Eastern (21.4%) provinces. Gender differences were observed, with males contributing a higher proportion of participants in Kigali City (45.1%) compared to females (31.7%).

In terms of insurance coverage, 9.2% of the participants reported having health insurance, with males being slightly more likely to have coverage (13.7%) compared to females (8.5%). Among those without insurance, females accounted for 91.5%, reflecting their predominance in the study population.

Regarding MH service utilization, 24.2% of participants were first-time users and 75.8% were repeat users. The proportions of first-time and repeat users were similar across genders, with 23.5% of males and 24.3% of females being first-time users, while 76.5% of males and 75.7% of females were repeat users.

This distribution highlights the predominance of females in the sample and their greater engagement with MH services, as well as geographic and age-related variations in demographic and service utilization characteristics.

Table 2 shows the frequencies and prevalences of first-time and repeat MH service utilization stratified by gender, age, province, and insurance coverage, with associated CIs and *p*-values. The prevalence of first-time MH service utilization was similar between females (24.3%, 95% CI: 21.3–27.6) and males (23.5%, 95% CI: 16.3–32.7). Repeat utilization rates were also comparable, with females at 75.7% (95% CI: 72.4–78.7) and males at 76.5% (95% CI: 67.3–83.7), with no statistically significant difference between genders (*p* = 0.858).

Age showed significant differences in utilization patterns (*p* < 0. 001). First-time utilization was highest among participants under 18 years (34.0%, 95% CI: 22.0–48.6) and 18–30 years (46.4%, 95% CI: 35.0–58.1). In contrast, repeat utilization was most common among participants aged 31–50 years (77.2%, 95% CI: 73.0–81.0) and over 50 years (79.4%, 95 percent CI: 73.3–84.4), indicating that older individuals were more likely to be repeat users.

Geographic variations were observed, with the highest prevalence of first-time utilization in Kigali City (28.1%, 95% CI: 23.1–33.8), followed by the Southern (23.9%, 95% CI: 18.6–30.2), Northern/Western (21.8%, 95% CI: 16.3–28.4), and Eastern (21.8%, 95% CI: 16.3–28.4) provinces. Repeat utilization followed a similar trend, with Kigali City at 71.9% (95% CI: 66.2–76.9), though differences across provinces were not statistically significant (*p* = 0.333).

Insurance coverage significantly influenced utilization patterns (*p* < 0.001). First-time utilization was more common among participants with insurance (45.2%, 95% CI: 33.3–57.6) compared to those without insurance (21.3%, 95% CI: 18.3–24.6). Conversely, repeat utilization was higher among uninsured participants (78.7%, 95% CI: 75.4–81.7) than among those with insurance (54.8%, 95% CI: 42. 4–66.7).

Table 3 presents the prevalence of insurance coverage stratified by gender, age, province, and MH service utilization. Overall, only 9.3% (95% CI: 7.4–11.6) of participants were insured, indicating limited health insurance coverage among the study population. Gender-specific results showed slightly higher insurance coverage among males (13.7%, 95% CI: 8.1–22.2) compared to females (8.5%, 95% CI: 6.6–10.9). However, insured rates remained low for both genders.

Age-specific trends revealed that participants over 50 years had the lowest insured prevalence at 4.8% (95% CI: 2.5–9.0). Younger participants under 18 years and 18–30 years had slightly higher insured rates of 11.9% (95% CI: 5.0–25.6) and 11.3% (95% CI: 5.5–21.9), respectively. Participants ages 31–50 years also showed limited insurance coverage, with a prevalence of 11.4% (95% CI: 8.5–15.1).

Geographic differences were notable, with Kigali City having the highest insured prevalence at 27.9% (95% CI: 21.7–35.1). In contrast, participants from other provinces had substantially lower insurance coverage—1.9% (95% CI: 0.7–4.9) in the Southern, 5.1% (95% CI: 2.6–9.4) in the Northern/Western, and 4.1% (95% CI: 2.0–8.3) in the Eastern provinces.

MH service utilization patterns showed higher insured prevalence among first-time users (16.9%, 95% CI: 11.9–23.4) compared to repeat users (6.2%, 95% CI: 4.5–8.6), suggesting that insurance may facilitate initial access to MH services.

These findings highlight consistently low insurance coverage across the population, with notable disparities by age, geographic location, and MH service utilization, particularly among older individuals and those residing outside Kigali City.

Table 4 displays the CORs and AORs with 95% CIs and *p*-values for factors associated with repeated MH service utilization.

Gender was not significantly associated with repeated service utilization. Compared to females, males had a COR of 1.1 (95% CI: 0.6–1.7, *p* = 0.858) and an AOR of 1.3 (95% CI: 0.7–2.4, *p* = 0.347).

Age showed significant associations in the adjusted model. Compared to participants under 18 years, those aged 31–50 years were significantly more likely to be repeat users (AOR: 2.3, 95% CI: 1.1–4.6, *p* = 0.022). Participants over 50 years also demonstrated increased odds of repeat utilization, though this association was marginally significant (AOR: 2.0, 95% CI: 1.0–4.3, *p* = 0.065). In contrast, participants ages 18–30 years had lower, though not statistically significant, odds of repeated utilization (AOR: 0.5, 95% CI: 0.2–2.2, *p* = 0.143).

Geographic location showed no significant associations in either the crude or adjusted models. Compared to participants from Kigali, those in the Southern (AOR: 0.9, 95% CI: 0.5–1.5, *p* = 0.602), Northern/Western (AOR: 1.0, 95% CI: 0.6–1.8, *p* = 0.923), and Eastern (AOR: 1.7, 95% CI: 0.9–3.1, *p* = 0.086) provinces showed no strong evidence of differing odds of repeated utilization.

Insurance coverage was strongly associated with repeated MH service utilization. Participants without insurance had significantly higher odds of repeated utilization compared to those with insurance, with an AOR of 3.3 (95% CI: 1.8–6.2, *p* < 0.001).

These findings underscore the role of insurance coverage and age in influencing repeated MH service utilization. Participants without insurance and those ages 31–50 years were more likely to engage in repeated utilization, while geographic and gender differences were not significant.

## 4. Discussion

The findings from this study provide a comprehensive view of the factors associated with repeat MH service utilization during Rwanda’s genocide commemoration week, revealing critical insights into the factors that influence ongoing MH support needs during this period of intense remembrance. A significant finding of this study is the high prevalence of repeat MH service users, comprising 76% of the total sample. This underscores the persistent and recurring nature of MH needs among individuals affected by the genocide, reflecting the long-term psychological impact of such traumatic events. The elevated rate of repeat utilization highlights the necessity for sustained MH interventions and support systems to address chronic MH conditions in this population.

Mental health service utilization during the genocide commemoration week follows a recurring pattern, with many individuals seeking support each year. The emotional intensity of this period often leads to distress, particularly among those with a history of trauma. The cyclical nature of mental health service utilization during the genocide commemoration week suggests that individuals are not only responding to personal distress but are also experiencing a collective psychological reactivation of trauma. Commemorative rituals, such as public testimonies, memorial visits, and communal mourning, serve as powerful emotional triggers that resurface traumatic memories, particularly for genocide survivors and their descendants [17]. This aligns with research indicating that exposure to reminders of past atrocities during such events can induce acute distress and re-traumatization. A study conducted in Rwanda found that 65% of respondents experienced trauma symptoms after the commemoration period, with 67% reporting multiple symptoms, including excessive anxiety, sadness, flashbacks, and hypervigilance [17]. The predictable rise in mental health service utilization during this period is, therefore, not solely a function of individual coping mechanisms but reflects broader collective trauma dynamics that necessitate tailored mental health interventions.

This study offers unique insights into repeat MH service utilization during the genocide commemoration week, addressing a critical gap in understanding long-term MH needs. By collecting data from health centers, district hospitals, and provincial facilities nationwide, it captures a representative sample across diverse regions and demographics. Additionally, the study highlights vulnerable groups, such as middle-aged individuals and those without insurance, providing valuable information to inform future interventions and policies targeting these populations.

The study faced several limitations, including the lack of prior research on repeat MH service use during Rwanda’s genocide commemoration week, limiting comparative analysis and broader conclusions. The absence of detailed data collection during follow-up visits restricts understanding of the changes in MH status over time, and financial constraints limit the number of these visits, impacting continuous support. Additionally, the inability to link walk-in visits to repeat service use complicates continuity-of-care tracking, and a gender and district imbalance in the sample may affect the generalizability of the findings. The intense emotional states of clients during the genocide commemoration week, along with the focus on immediate support, potentially impacted data completeness and reduced available variables for analysis, limiting the study’s depth.

Gender did not emerge as a significant factor influencing repeat MH service utilization, with both males and females showing similar patterns of repeat use. This finding aligns with the broader literature that emphasizes the universal impact of traumatic events on MH across genders [14]. Age emerged as a notable factor, especially for middle-aged individuals (31–50 years), who demonstrated significantly higher odds of being repeat users compared to younger individuals. This finding suggests that middle-aged adults may face more severe or persistent MH challenges related to the genocide, underscoring a need for ongoing support. As reported in previous research, older individuals showed higher prevalence rates of PTSD and other MH disorders, which may be due to cumulative life stressors and prolonged trauma exposure [18]. The significant impact of age on service utilization mirrors studies that highlight the cumulative effect of trauma over the lifespan, particularly among middle-aged adults who lived through the genocide [18].

Geographical location, as indicated by the provinces of service within Rwanda, does not significantly impact the likelihood of repeat MH service use, reflecting the widespread availability of these services and the uniform impact of the genocide across different regions. However, as suggested by Eichelsheim, subtle regional variations might still exist, which were not captured in this study, due to the broad categorization of provinces [19].

Individuals without insurance were significantly more likely to be repeat users of MH services. A lack of insurance is a critical barrier to accessing and maintaining MH care. Uninsured individuals may face financial hardships that necessitate the repeated use of available MH services during periods of crisis, such as the genocide commemoration week. Sabey et al. [20] reported that while decentralization and integration strategies increased care accessibility in a post-genocide Rwanda, a more collaborative, adaptive approach is essential to address local needs effectively and ensure equitable access to MH care. Persistent demand for MH services was reported previously by Rieder et al. [14] and Kagoyire et al. [13], who documented the long-term psychological effects of the genocide on survivors and their descendants. The critical barrier posed by a lack of insurance coverage resonates with broader findings on the socioeconomic determinants of health and the importance of financial accessibility in maintaining MH care [20].

To strengthen mental health service delivery during Rwanda’s genocide commemoration week, targeted and culturally responsive interventions should be implemented. First, enhancing data collection during follow-up sessions by incorporating standardized assessments of trauma severity and social support can provide deeper insights into repeat service utilization patterns. This would enable early identification of high-risk individuals who require sustained support beyond the commemoration period.

Second, leveraging Rwanda’s extensive network of community health workers (CHWs) and local opinion leaders to provide trauma-sensitive psychological first aid can increase accessibility, particularly in rural areas where formal mental health services are limited. Training CHWs to conduct basic trauma screenings and refer high-risk individuals would help integrate mental health support into primary care settings.

Third, Rwanda’s health system could implement a structured referral system that ensures individuals identified with severe psychological distress during the commemoration week receive continued care beyond the event, preventing crisis recurrence each year. This could involve scaling up telemedicine options for follow-up care, particularly in regions with fewer mental health professionals.

Finally, targeted outreach strategies for males and younger populations should be developed to address the unique mental health barriers they face. This could include integrating mental health discussions into youth programs, faith-based gatherings, and workplace wellness initiatives, ensuring broader engagement of populations that may otherwise hesitate to seek formal services.

This study is cross-sectional and relies on binary logistic regression, which identifies associations but does not track changes in mental health status over multiple years. While this approach is useful for detecting factors associated with repeat service utilization during the genocide commemoration week, it does not capture the full trajectory of mental health needs over time. Additionally, the dataset does not include longitudinal records, as systematic individual-level documentation of service utilization was only initiated in 2024.

However, as more records become available in future years, longitudinal tracking may provide deeper insights into evolving trauma responses and mental health service needs. Future research should build on this by examining urban–rural disparities in access to mental health services, investigating the needs of younger generations affected by intergenerational trauma, and incorporating longitudinal methods to monitor service utilization trends over time. Expanding research to include qualitative or mixed-methods approaches could also help capture personal experiences and motivations for seeking mental health care during this period.

Furthermore, collaboration with government agencies and NGOs will be essential to secure funding and support for comprehensive follow-up programs that ensure sustained mental health interventions beyond the commemoration week.

## 5. Conclusions

This study highlights the significant and persistent need for MH services among individuals affected by Rwanda’s 1994 genocide, particularly during the annual genocide against Tutsi commemoration week. The high prevalence of repeat service utilization, especially among middle-aged individuals and those without insurance, underscores the ongoing psychological impact and the barriers to accessing consistent MH care. These findings align with broader research on the long-term effects of trauma and the critical role of socioeconomic factors in MH service utilization. Addressing these needs through targeted interventions and improving healthcare or insurance coverage can enhance MH support systems, promoting resilience and well-being in the Rwandan community. This study contributes valuable insights for policymakers and healthcare providers, emphasizing the importance of comprehensive and accessible MH care during periods of intense national remembrance.

## Figures and Tables

**Table 1:** Demographic Characteristics of Individuals Seeking Mental Health Services During the 2024 Genocide Commemoration Week (N = 825).

		Males		Females		Both	
		*n*	%	*n*	%	*n*	%
Age	<18	7	6.7	41	6.1	48	6.2
	18–30	10	9.6	60	8.9	70	9
	31–50	64	61.5	383	56.7	447	57.3
	>50	23	22.1	192	28.4	215	27.6
Province	Kigali	50	45.1	241	31.7	291	33.4
	Southern	24	21.6	191	25.1	215	24.7
	Northern/Western	16	14.4	163	21.4	179	20.6
	Eastern	21	18.9	165	21.7	186	21.4
Insurance coverage	Yes	13	13.7	54	8.5	67	9.2
	No	82	86.3	581	91.5	663	90.8
Mental health service utilization	First-time user	24	23.5	176	24.3	200	24.2
	Repeat user	78	76.5	547	75.7	625	75.8

**Table 2:** Frequencies and Prevalences of First-Time and Repeat Mental Health Service Utilization by Key Variables During the 2024 Genocide Commemoration Week (N = 825).

		First Time User (n)	% (95% CI)	Repeat User (n)	% (95% CI)	*p*-Value
Gender	Female	176	24.3 (21.3–27.6)	547	75.0 (72.4–78.7)	0.858
	Male	24	23.5 (16.3–32.7)	78	76.5 (67.3–83.7)	
Age	<18	16	34.0 (22.0–48.6)	31	66.0 (51.4–78.0)	0.000
	18–30	32	46.4 (35.0–58.1)	37	53.6 (41.9–65.0)	
	31–50	98	22.8 (19.1–27.0)	332	77.2 (73.0–81.0)	
	>50	42	20.6 (15.6–26.7)	162	79.4 (73.3–84.4)	
Province	Kigali	76	28.1 (23.1–33.8)	194	71.9 (66.2–76.9)	0.333
	Southern	49	23.9 (18.6–30.2)	156	76.1 (69.8–81.4)	
	Northern/Western	39	21.8 (16.3–28.4)	140	78.2 (71.6–83.7)	
	Eastern	39	21.8 (16.3–28.4)	140	78.2 (71.6–83.7)	
Insurance coverage	Yes	28	45.2 (33.3–57.6)	34	54.8 (42.4–66.7)	0.000
	No	138	21.3 (18.3–24.6)	511	78.7 (75.4–81.7)	

**Table 3:** Health Insurance Coverage Among Individuals Seeking Mental Health Services During the 2024 Genocide Commemoration Week, Stratified by Key Variables (N = 825).

Variable		Insured% (95% CI)	Uninsured% (95% CI)
Gender	Female	8.5 (6.6–10.9)	91.5 (89.1–93.4)
	Male	13.7 (8.1–22.2)	86.3 (77.8–91.9)
Age	<18	11.9 (5.0–25.6)	88.1 (74.4–95.0)
	18–30	11.3 (5.5–21.9)	88.7 (78.1–94.5)
	31–50	11.4 (8.5–15.1)	88.6 (84.9–91.5)
	>50	4.8 (2.5–9.0)	95.2 (91.0–97.5)
Province	Kigali City	27.9 (21.7–35.1)	72.1 (65.0–78.3)
	Southern	1.9 (0.7–4.9)	98.1 (95.1–99.3)
	Northern/Western	5.1 (2.6–9.4)	94.9 (90.6–97.4)
	Eastern	4.1 (2.0–8.3)	95.9 (91.7–98.0)
Mental health service utilization	First-time user	16.9 (11.9–23.4)	83.1 (76.6–88.1)
	Repeat user	6.2 (4.5–8.6)	93.8 (91.4–95.5)

**Table 4:** Crude and Adjusted Odds Ratios for Factors Associated with Repeat Mental Health Service Utilization During the 2024 Genocide Commemoration Week.

		Crude Odds Ratios (COR)	95% CI	*p*-Value	Adjusted Odds Ratios (AOR)	95% CI	*p*-Value
Gender	Female	1	··	··	1	··	··
	Male	1.1	0.6–17	0.858	1.3	0.7–2.4	0.347
Age	<18	1	··	··	1	··	··
	18–30	0.6	0.3–1.3	0.187	0.5	0.2–2.2	0.143
	31–50	1.8	0.9–3.3	0.089	2.3	1.1–4.6	0.022
	>50	2.0	1.0–4.0	0.051	2.0	1.0–4.3	0.065
Province	Kigali	1	··	··	1	··	··
	Southern	1.2	0.8–1.9	0.298	0.9	0.5–1.5	0.602
	Northern/Western	1.4	0.9–2.2	0.131	1	0.6–1.8	0.923
	Eastern	1.4	0.9–2.2	0.131	1.7	0.9–3.1	0.086
Insurance	Yes	··	··	··	1	··	··
	No	3	1.8–5.2	0.000	3.3	1.8–6.2	0.000

## Data Availability

The datasets used and analyzed during the current study are available from the corresponding author upon reasonable request. The data are not publicly available due to privacy reasons.

## Abbreviations

The following abbreviations are used in this manuscript:

PTSD	Post Traumatic Stress Disorder
RBC	Rwanda Biomedical Centre
COR	Crude odds ratios
AOR	Adjusted Odds Ratios
MH	Mental Health
CI	Confidence Interval
